# Supplementary material for: Reorganization of the flagellum scaffolding induces a sperm standstill during fertilization
Source: eLife. 2024 Nov 13;13:RP93792. doi: 10.7554/eLife.93792 (PMC11560130; doi:10.7554/eLife.93792)
Supplement: MDAR checklist [file elife-93792-mdarchecklist1.docx]

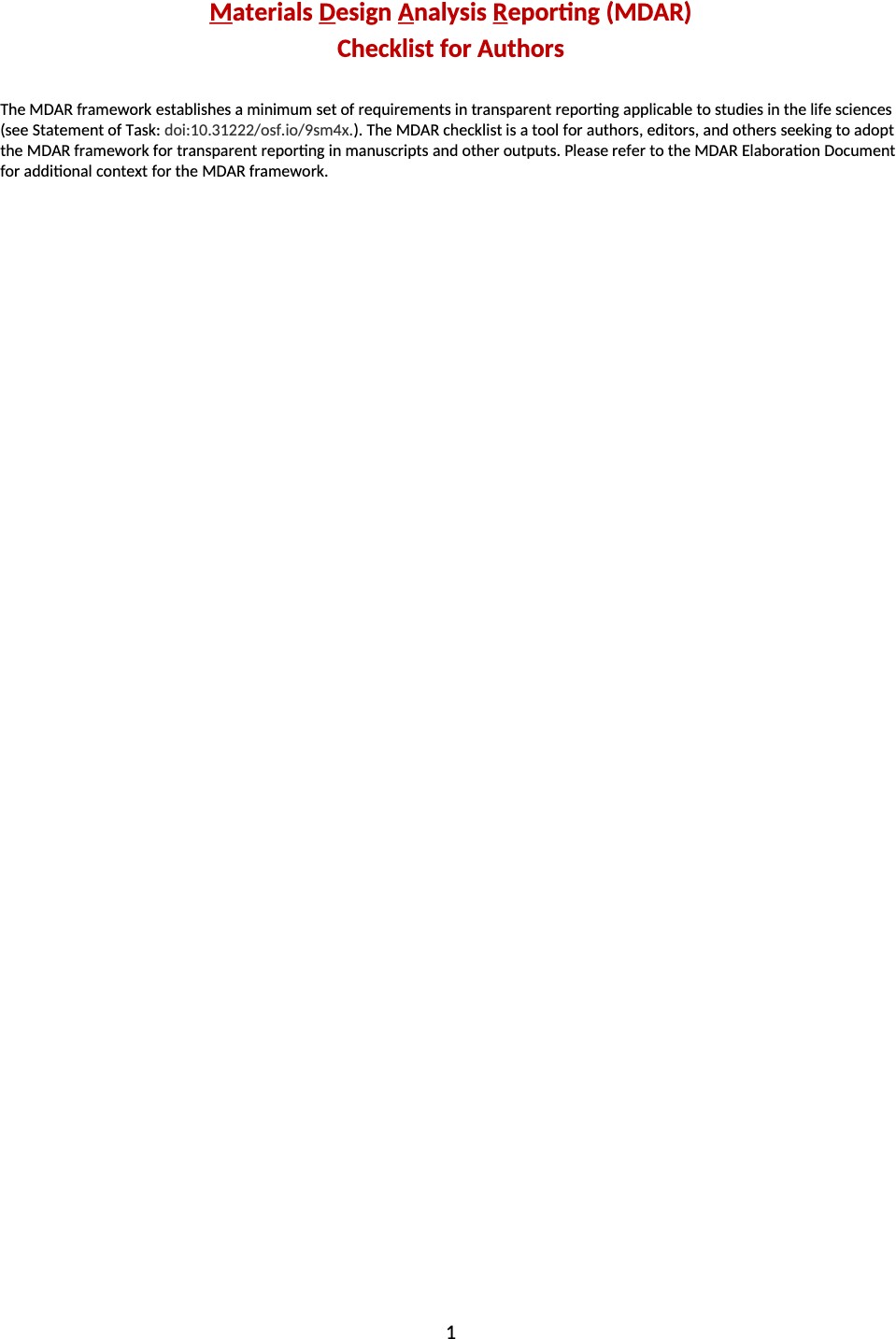


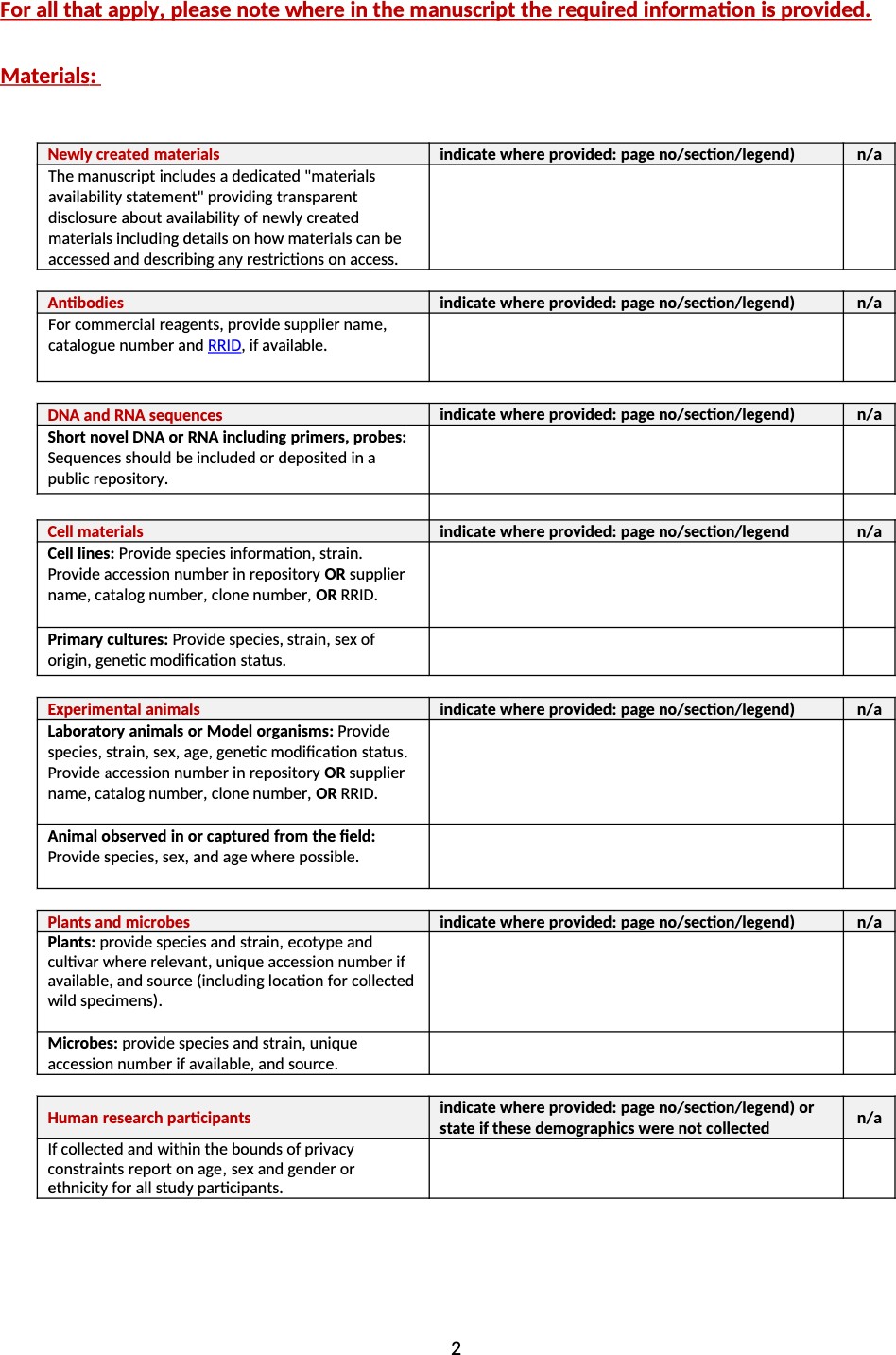


N/A

N/A

N/A

Yes - see the Materials and Methods section entitled “Reagents and chemical sources”.

Yes - see the Materials and Methods section entitled “Animals and housing conditions”.

N/A

N/A

N/A

N/A

N/A


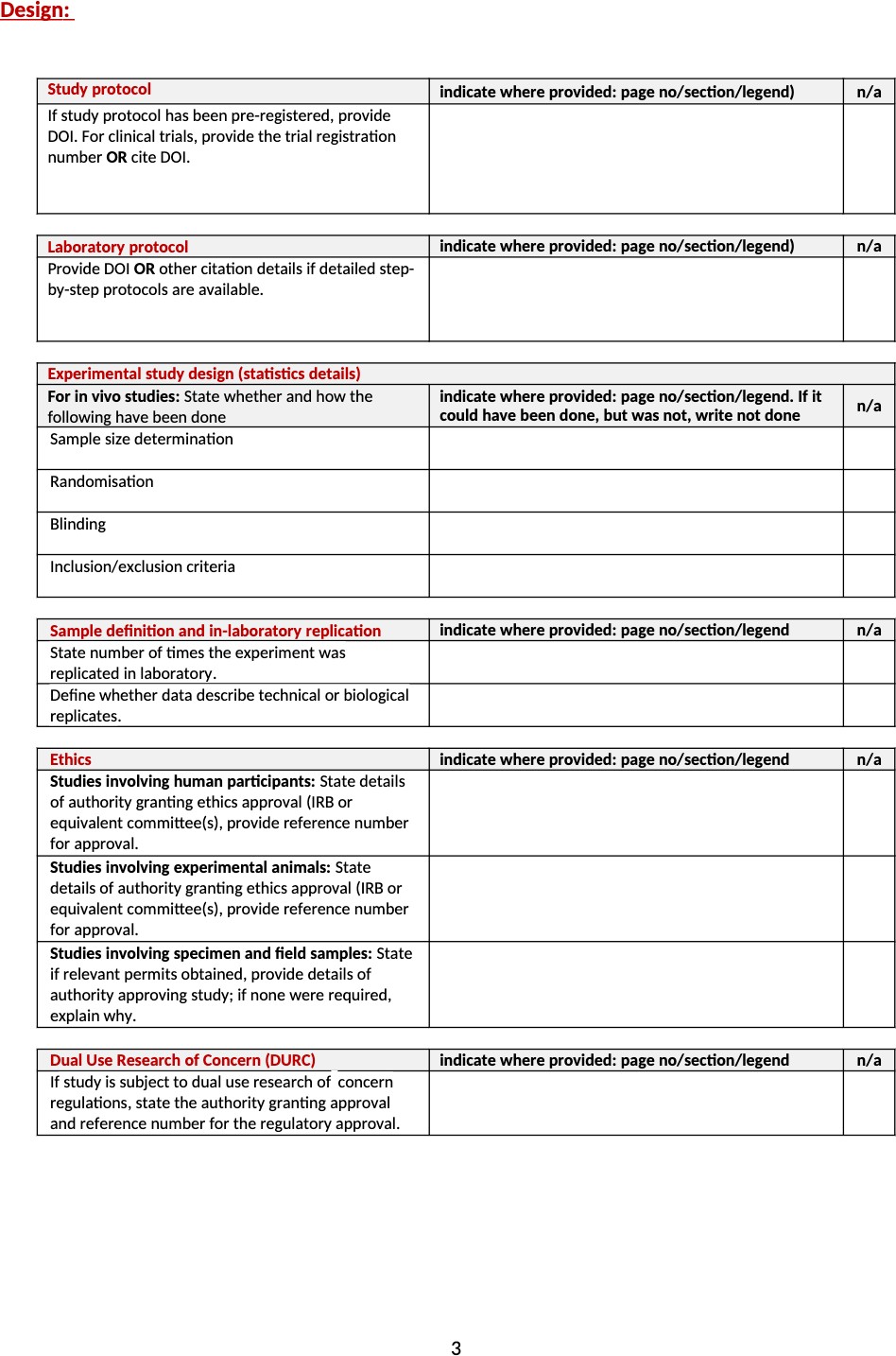


See the Materials and Methods section entitled “Animals and housing conditions”.

Experiments were replicated at least 3 times. Sample sizes are shown in Tables and Figure legends, and represent biological replicates (individual cells or animals).

Data describe biological replicates (individual cells or animals).

N/A

N/A

N/A

N/A

N/A

N/A

N/A

N/A

N/A


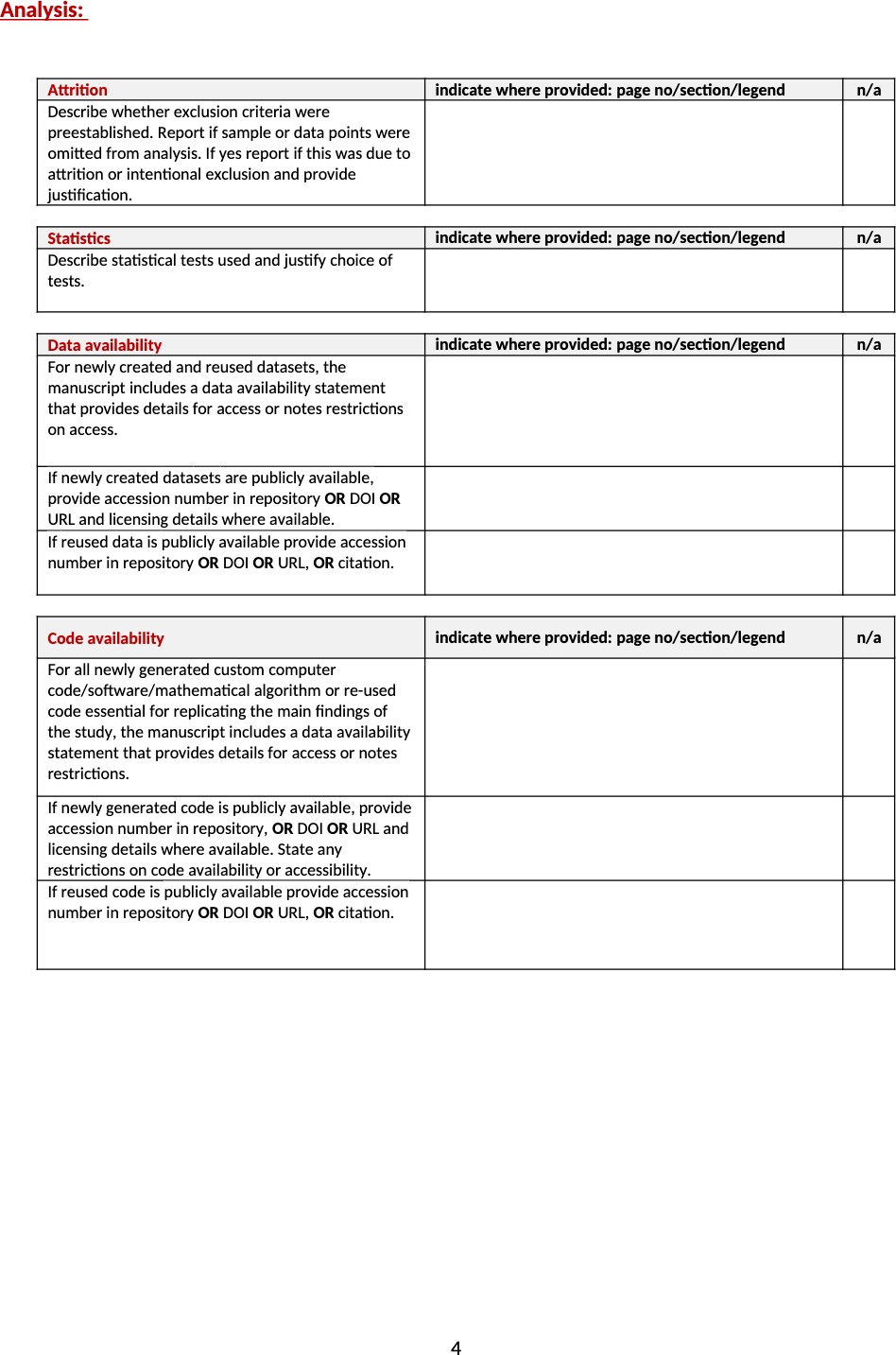


Github

<https://github.com/martijab/Reorganization-of-the-Flagellum-Scaffolding-Induces-a-Sperm-Standstill-During-Fertilization.git>

Github

<https://github.com/martijab/Reorganization-of-the-Flagellum-Scaffolding-Induces-a-Sperm-Standstill-During-Fertilization.git>

Zenodo. <https://doi.org/10.5281/zenodo.13769608>

Zenodo. <https://doi.org/10.5281/zenodo.13769608>

See the Materials and Methods section entitled “Statistical Analysis”.

N/A

N/A

N/A


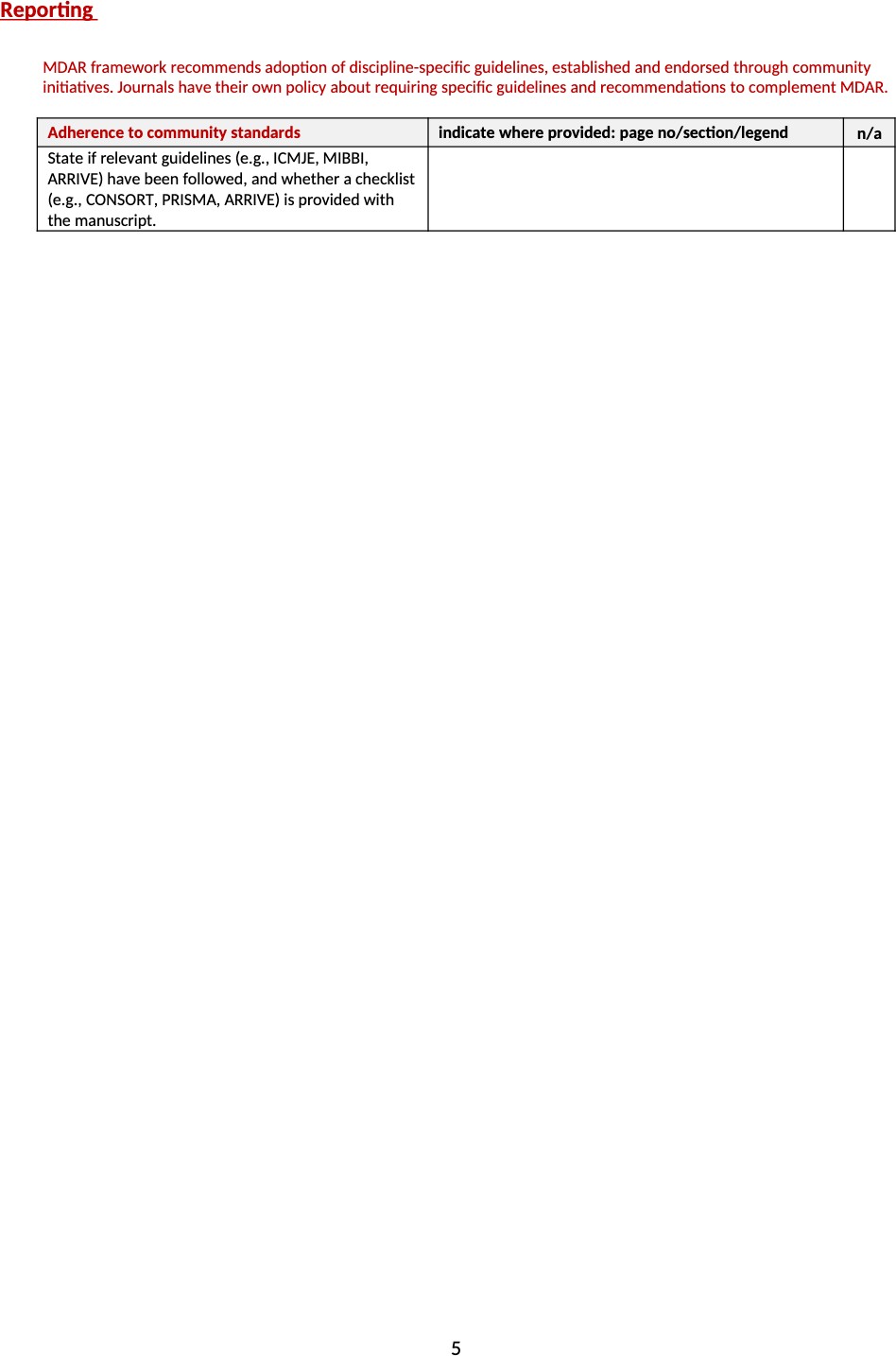


N/A
